# Identification of disease-related genes and construction of a gene co-expression database in non-alcoholic fatty liver disease

**DOI:** 10.3389/fgene.2023.1070605

**Published:** 2023-03-27

**Authors:** Hua Ye, Mengxia Sun, Mingli Su, Dahua Chen, Huiwei Liu, Yanyan Ma, Wenjing Luo, Hong Li, Feng Xu

**Affiliations:** ^1^ Department of Gastroenterology, The Affiliated Lihuili Hospital, Ningbo University, Ningbo, Zhejiang, China; ^2^ Department of Clinical Medicine, Health Science Center, Ningbo University, Ningbo, Zhejiang, China; ^3^ Department of Hepatobiliary Surgery, The Affiliated Lihuili Hospital, Ningbo University, Ningbo, Zhejiang, China

**Keywords:** module eigengene, M6A, connectivity, differential module, ROC

## Abstract

**Background:** The mechanism of NAFLD progression remains incompletely understood. Current gene-centric analysis methods lack reproducibility in transcriptomic studies.

**Methods:** A compendium of NAFLD tissue transcriptome datasets was analyzed. Gene co-expression modules were identified in the RNA-seq dataset GSE135251. Module genes were analyzed in the R gProfiler package for functional annotation. Module stability was assessed by sampling. Module reproducibility was analyzed by the ModulePreservation function in the WGCNA package. Analysis of variance (ANOVA) and Student’s t-test was used to identify differential modules. The receiver operating characteristic (ROC) curve was used to illustrate the classification performance of modules. Connectivity Map was used to mine potential drugs for NAFLD treatment.

**Results:** Sixteen gene co-expression modules were identified in NAFLD. These modules were associated with multiple functions such as nucleus, translation, transcription factors, vesicle, immune response, mitochondrion, collagen, and sterol biosynthesis. These modules were stable and reproducible in the other 10 datasets. Two modules were positively associated with steatosis and fibrosis and were differentially expressed between non-alcoholic steatohepatitis (NASH) and non-alcoholic fatty liver (NAFL). Three modules can efficiently separate control and NAFL. Four modules can separate NAFL and NASH. Two endoplasmic reticulum related modules were both upregulated in NAFL and NASH compared to normal control. Proportions of fibroblasts and M1 macrophages are positively correlated with fibrosis. Two hub genes *Aebp1* and *Fdft1* may play important roles in fibrosis and steatosis. m6A genes were strongly correlated with the expression of modules. Eight candidate drugs for NAFLD treatment were proposed. Finally, an easy-to-use NAFLD gene co-expression database was developed (available at https://nafld.shinyapps.io/shiny/).

**Conclusion:** Two gene modules show good performance in stratifying NAFLD patients. The modules and hub genes may provide targets for disease treatment.

## Introduction

Due to the drastically changed living style in modern life, non-alcoholic fatty liver disease (NAFLD) has become an epidemic and imposes a heavy burden on public health. NAFLD encompasses a series of progressive liver diseases developing from simple steatosis (NAFL) to hepatocyte cell death (ballooning) and inflammation (non-alcoholic steatohepatitis, NASH) (Lefebvre et al., 2017). NAFL is generally regarded as a reversible benign condition (Govaere et al., 2020). Mitochondrion plays a role in the phenotypic switching from NAFL to NASH(Pirola et al., 2013). Patients with NASH may progress to cirrhosis and hepatocellular carcinoma (HCC) (Arendt et al., 2015). Although not a prerequisite for diagnosis, fibrosis can also occur and is associated with adverse outcomes (Kozumi et al., 2021).

Transcriptomics is a powerful tool to investigate the expression of thousands of genes concurrently (Liu and Wang, 2020). Large-scale transcriptome data is valuable for developing diagnostic biomarkers, as well as for targeting therapy (Sookoian and Pirola, 2020). There is currently no approved therapy for non-alcoholic steatohepatitis (NASH). The systems biology method may help to dissect the disease mechanisms and to translate basic research into useful treatments (Sookoian and Pirola, 2019). The transcriptome of NAFLD patients has been profiled in several studies (Ahrens et al., 2013; Murphy et al., 2013; Arendt et al., 2015; Hoang et al., 2019; Azzu et al., 2021; Pantano et al., 2021). Some of the studies are based on the microarray, while some are based on state-of-the-art RNA-Seq technology. However, to what extent the results of these studies are reproducible is still not known. A systematic meta-analysis of microarray experiments on liver tissue of NAFLD patients has been performed, and four genes were identified as biomarkers for patients at risk of progression to severe NAFLD (Ryaboshapkina and Hammar, 2017). The study lacks RNA-Seq data. Only one recent report showed that four genes were consistently identified by all six NAFLD transcriptome studies (Pantano et al., 2021). The reason for the phenomenon may include different sample sizes, control samples chosen, platforms used, and data processing methods used (Ye and Liu, 2015).

As it is well known that complex human diseases such as NAFLD and cancer are rarely caused by a single gene but are more likely influenced by a network of interacting genes (Sookoian et al., 2020; Paci et al., 2021). Genetic redundancy accounts that a given biochemical function is redundantly encoded by two or more genes. Therefore, mutations (or defects) in one of these genes will have a smaller effect on the fitness of the organism than expected from the genes’ function (Pearce et al., 2004). Differential gene lists from individual studies often lack reproducibility. Module-level differential analysis may help to overcome problem. A gene co-expression module is a more stable unit than a single gene in diseases (Zhou et al., 2019). An example is cancer where the aberrant cell cycle is recurrently present, but the change of a cell cycle-related gene may present in one dataset but not another (Zhou et al., 2019). The rationale of gene co-expression analysis is that gene expressions are correlated. Weighted gene co-expression network analysis (WGCNA) can reduce thousands of genes to tens of modules, which are relatively independent as genes in a module have similar expression patterns but are different from that of other modules. Thus, a module may represent a unique function of a bio-system. Therefore, it is reasonable to reanalyze these valuable datasets by gene co-expression network.

**FIGURE 1:**
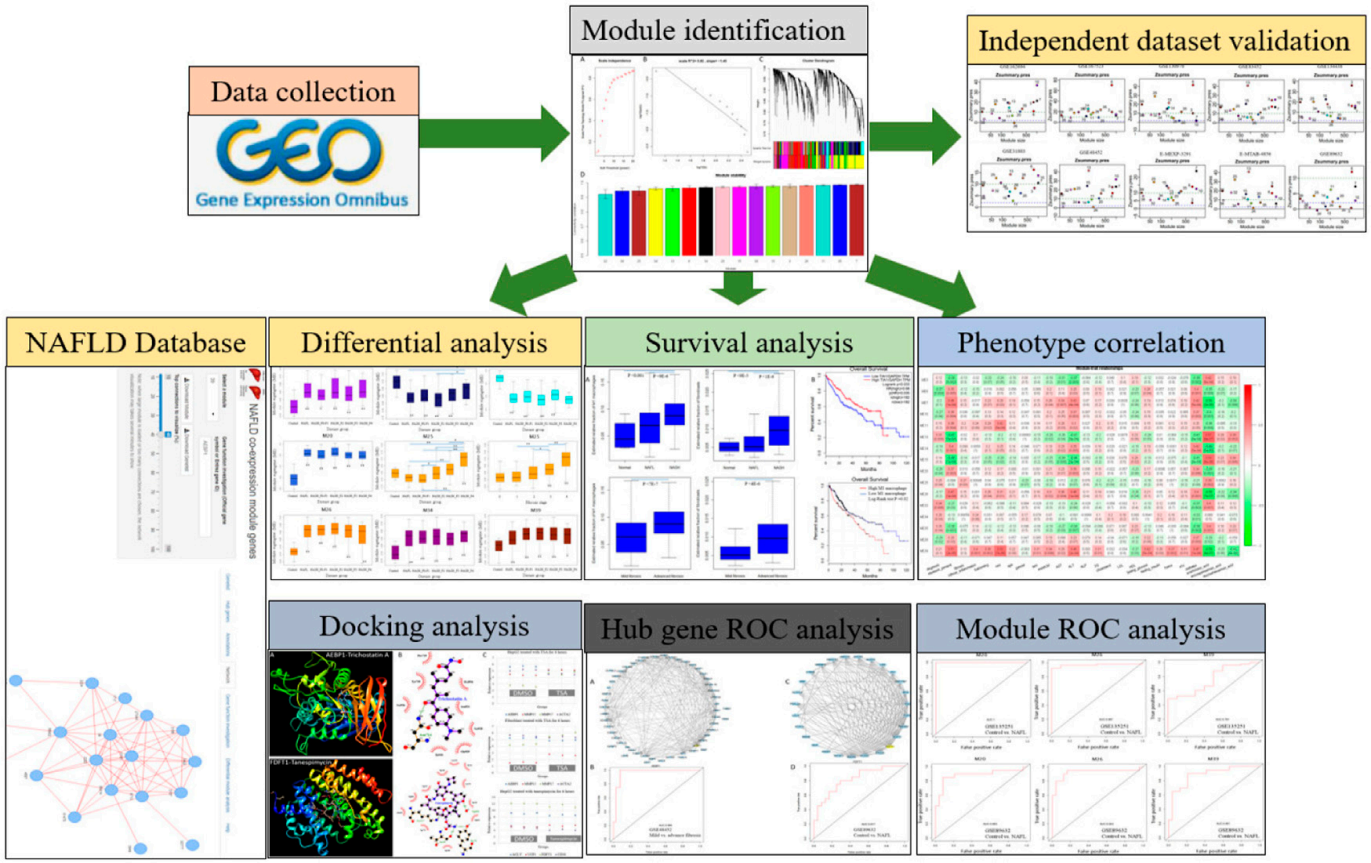
A schematic diagram for the analysis framework. NAFLD transcriptome datasets were collected and analyzed by gene co-expression network analysis. Independent datasets were used for network validation. Based on the identified network modules, the NAFLD database was constructed. Module-level differential analysis, ROC analysis, and phenotype correlation were performed. Module hub genes were mined for potential drug discovery.

Here, we first applied WGCNA to the currently largest NAFLD tissue dataset GSE135251 which was profiled by RNA-Seq. Identified modules were used as a reference, where new datasets can be projected, making the comparisons between datasets possible. We found a sterol biosynthesis module and a collagen-containing extracellular matrix module. Both modules can efficiently and recurrently separate patients of NAFLD in different datasets. Proportions of fibroblasts and M1 macrophages are positively correlated with fibrosis. Eight candidate drugs were also identified based on the hub genes of differential modules. The analysis workflow is summarized in Figure 1.

## Materials and methods

### Datasets and preprocessing

A total of 11 NAFLD tissue datasets were downloaded from the National Center for Biotechnology Information (NCBI) Gene Expression Omnibus (GEO) and the European Bioinformatics Institute (EBI) ArrayExpress. These datasets were listed in Table 1. Among these datasets, four are from RNA-Seq and seven are from the microarray. GSE135251 is currently the largest NAFLD dataset available from public repositories, which contains 216 NAFLD samples across the disease spectrum. To focus on the informative genes, the count matrix provided by the database was filtered with a mean count >200 and a standard deviation >0.1 before downstream analysis. Finally, 7,773 genes were retained for gene co-expression network analysis. Additional two datasets (GSE200186 and GSE119281) were used for drug targets validation. As datasets listed in Table 1 are from different studies and platforms and may have different clinical/histological information available, these data were analyzed individually not in a pooled form.

**TABLE 1:** Datasets used in the study.

Accession	Sample	Platform	Clinical parameters
GSE135251	206 NAFLD, 10 controls	Illumina NextSeq 500	NAS, fibrosis
GSE162694	143 NASH patients of various fibrosis stages	Illumina HiSeq 3,000	Age, sex, fibrosis, NAS
GSE167523	98 NAFLD patients	Illumina HiSeq 3,000	Disease subtype, age, gender
GSE130970	72 NAFLD, 6 control	Illumina HiSeq 2,500	Sex, age, lobular inflammation grade, cytological ballooning grade, steatosis grade, NAS, fibrosis
GSE83452	126 NASH samples, 66 no NASH	Affymetrix Human Gene 2.0 ST Array	Age, gender
GSE134438	43 NAFLD patients	Affymetrix Human Gene 2.0 ST Array	Disease subtype
GSE49541	72 NAFLD samples	Affymetrix Human Genome U133 Plus 2.0 Array	Fibrosis
GSE48452	32 NAFLD, 41 control	Affymetrix Human Gene 1.1 ST Array	Fat, inflammation, sex, age, BMI, NAS, fibrosis, leptin, adiponectin
E-MEXP-3291	26 NAFLD samples, 19 control	Affymetrix GeneChip Human Gene 1.0 ST Array	Age, sex, diagnosis
E-MTAB-4856	88 NAFLD samples	Agilent Whole Human Genome Microarray 4 × 44K 014,850 G4112F	Age, sex, disease staging, BMI
GSE89632	29 NAFLD, 24 control	Illumina HumanHT-12 WG-DASL V4.0 R2 expression beadchip	Diagnosis, steatosis, fibrosis, lobular inflammation, ballooning, NAS, age, gender, BMI, waist, AST, ALT, ALP, TG, TC, LDL, HDL, FPG, fasting insulin, HOMA-IR, HbA1c, diabetes

NAS: NAFLD, activity score, BMI: body mass index, AST: aspartate transaminase, ALT: alanine transaminase, ALP: alkaline phosphatase, TG: triglycerides, TC: total cholesterol, LDL: low-density lipoprotein cholesterol, HDL: high-density lipoprotein cholesterol, FPG: fasting plasma glucose, HOMA-IR: homa-insulin resistance, HbA1c: hemoglobin a1c.

### Weighted gene co-expression network analysis (WGCNA)

Gene co-expression module identification was performed according to the package manual (Langfelder and Horvath, 2008). Parameters were set as following: softPower = 16, corOptions = list (use = ‘p', method = ‘spearman’), networkType = “signed”, minModuleSize = 30, deepSplit = 4, MEDissThres = 0.2. Briefly, the pairwise Spearman correlation coefficient was calculated for each gene in the gene expression matrix, and then an adjacency matrix was derived by raising the correlation matrix to a power 16, which generated a biologically meaningful scale-free network. The weighted network was transformed into a network of topological overlap (TO)—a metric that defines the relationship of two genes accounting for their correlation and shared neighborhood. Genes were hierarchically clustered based on their TO. Finally, co-expression gene modules were identified by the Dynamic Tree Cut algorithm. As genes in a module are highly correlated, module genes can be reduced to a module eigengene (ME) by singular value decomposition. ME represents the first principal component of module expression profiles (Zhang and Horvath, 2005). WGCNA also provides gene connectivity information, which is the sum of correlations of a gene with all other genes in the module or network. Hub gene in a co-expression module tends to have high connectivity and may play important roles in the network or module. For network module validation, the expression matrix was first intersected with the reference dataset, then its values were transformed to ranks before module projection. Module stability was tested by 1,000 half-samplings for each module (Liu et al., 2018a). The stability was presented by the correlation of intra-module connectivity between the original one and the sampled one in form of mean ± standard deviation. To test the reproducibility of these modules, other datasets were projected to the frozen reference for module preservation analysis (Langfelder et al., 2011). Parameters for module preservation were set as networkType = “signed”, nPermutations = 100. The module-level expression for other datasets was retrieved by the moduleEigengenes function.

### Functional annotation of the modules

The gProfileR package was used for enrichment analysis of reference modules (Reimand et al., 2016). For drug screening, hub genes of differential modules were submitted to the Connectivity Map (https://portals.broadinstitute.org/cmap) (Lamb et al., 2006). Significant results were retrieved at the level of *p* < 0.05. Protein-ligand docking was performed in SwissDock (http://www.swissdock.ch/docking) (Grosdidier et al., 2011). LigPlot+ was used to generate a pose view of protein-ligand interaction (Laskowski and Swindells, 2011). The proportion of immune cell populations was estimated by TIMER2.0 (http://timer.comp-genomics.org/) (Li et al., 2020). Immunohistochemistry images for hub genes *Aebp1* and *Fdft1* were retrieved from the Human Protein Atlas database (https://www.proteinatlas.org/). Survival analysis for genes and immune cells was retrieved from the GEPIA2021 database (http://gepia2021.cancer-pku.cn/).

### Statistical analysis

Differential module analyses were performed using the Student’s t-test or ANOVA in R package aov. Tukey’s HSD was used to calculate *p* values of multiple pairwise comparisons. An adjusted *p*-value smaller than 0.05 was considered statistically significant.

## Results

### Sixteen modules were identified in the NAFLD

**FIGURE 2:**
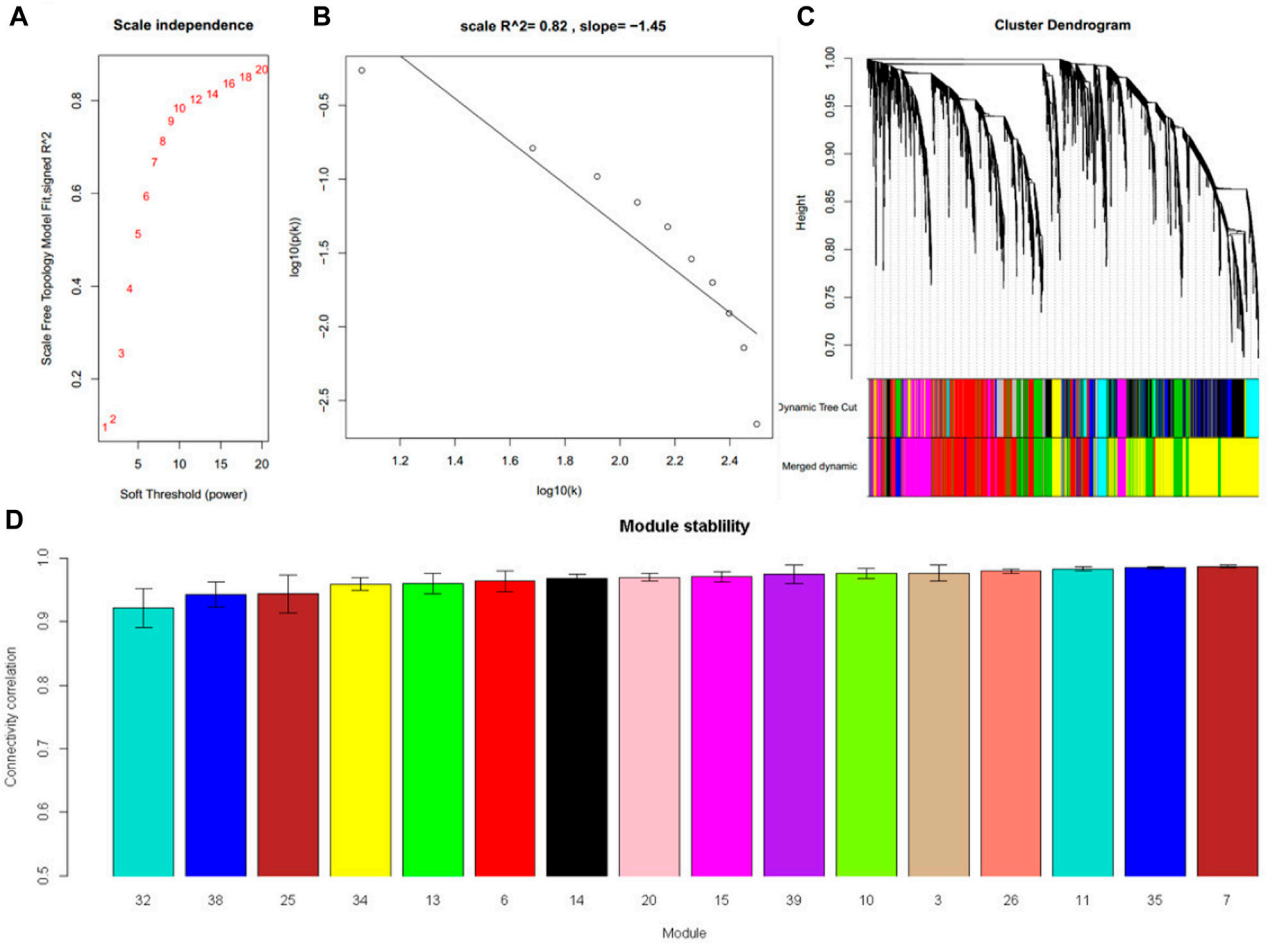
Sixteen modules were identified in the NAFLD dataset GSE135251. **(A)** The relationship between choosing power and corresponding scale-free topology model fit *R* ^2^ **(B)** When power was set at 16, the constructed network follows a power law. **(C)** Cluster dendrogram shows the partition of genes into co-expressed modules with different colors **(D)** The module stability was tested by sampling half of the samples. The stability was expressed as the correlation of intramodule connectivity between the original one and the sampled one.

RNA-Seq dataset GSE135251 was used to construct a frozen reference network, to which other datasets would be projected. A scale-free network was constructed (Figure 2A, B), and then a total of 16 gene co-expression modules were identified (Figure 2C). The top hub gene with high connectivity for each module was provided in Table 2. Functional annotation shows that these modules were associated with nucleus, translation, transcription factors, vesicle, immune response, mitochondrion, collagen, and sterol biosynthesis (Table 2). For a full list of genes, their assigned modules, connectivity, and gene description, readers can refer to Supplementary Table S1.

**TABLE 2:** Functional enrichment analysis for the sixteen modules identified in the NAFLD dataset GSE135251.

Module (No. genes)	Function (*p*-value)	Hub	TIMER cell type with highest correlation
3 (813)	Nucleus (2E-30) Factor: E2F (9E-15)	*Taf1c*	Memory CD4 T cells
6 (813)	Ribosome (3E-92) Translation (2E-81)	*Ndufa2*	Macrophage
7 (2,410)	Factor: HDAC2 (1E-61) hsa-miR-21–5p (2E-54)	*Tmem106b*	Th1 CD4 T cell (−)
10 (1,621)	Factor: Churchill (8E-76) Factor: Sp1 (1E-75)	*Anapc2*	Th1 CD4 T cell
11 (205)	Vesicle (2E-13) Mitochondrion (5E-13)	*Tm9sf2*	Memory CD4 T cells (−)
13 (171)	Immune response (1E-21) Myeloid leukocyte activation (3E-16)	*Mpeg1*	Macrophage
14 (170)	Mitochondrion (5E-13) Factor: ER81 (2E-11)	*Rbis*	Lymphoid progenitor
15 (153)	Nucleus (8E-17) RNA metabolism (1E-15)	*Tia1*	M1 Macrophage (−)
20 (337)	Vesicle (2E-18) Endoplasmic reticulum (5E-11) Sp1 (3E-10) hsa-miR-484 (5E-10)	*Clptm1*	Lymphoid progenitor (−)
25 (69)	Collagen-containing extracellular matrix (1E-26)	*Aebp1*	Fibroblast
26 (234)	Endomembrane system (6E-9) Endoplasmic reticulum (2E-4)	*Kctd20*	Macrophage (−)
32 (54)	Leukocyte activation involved in immune response (7E-8)	*Stk10*	Microenvironment score^*^
34 (92)	Nucleoplasm (3E-6)	*Trrap*	Lymphoid progenitor (−)
35 (386)	hsa-miR-21–5p (1E-12) Factor: ETF (2E-9)	*Dennd4c*	Granulocyte monocyte progenitor
38 (33)	Translation (1E-11) Structural constituent of ribosome (3E-10) Mitochondrial inner membrane (7E-10)	*Stoml2*	CD4 T cell (−)
39 (30)	Sterol biosynthesis (8E-39) Factor: YB-1 (1E-5)	*Fdft1*	

(−), the estimated fraction of cell type is negatively correlated with module eigengene (ME); All the correlations in the “TIMER, cell type with highest correlation” column are >0.6 and *p* < 0.01.

^a^Microenvironment score, the sum of all immune and stromal cell types.

**FIGURE 3:**
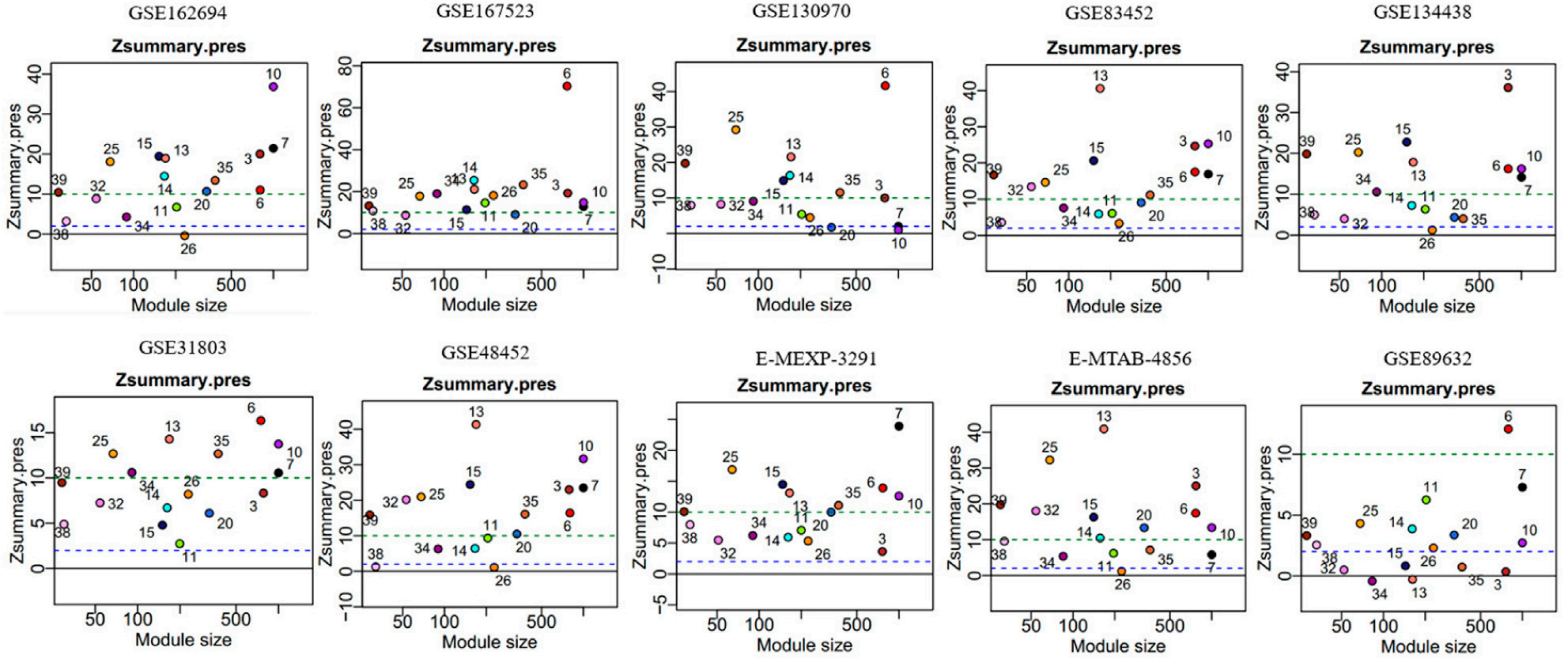
Gene network modules from GSE135251 are well preserved in the other ten datasets. The *y*-axis represents preservation statistics and the *x*-axis is the number of genes in each module. The dashed blue and green lines indicate the thresholds Z = 2 and Z = 10, respectively. Zsummary <2 implies no evidence for module preservation, 2 < Zsummary <10 implies weak to moderate evidence, and Zsummary >10 implies strong evidence for module preservation.

In module reproducibility analysis, all the modules had an average connectivity correlation larger than 0.9 (Figure 2D). Ten NAFLD datasets from different platforms (Illumina, Affymetrix, and Agilent in Table 1) were projected on the frozen reference modules to test reproducibility (Figure 3). All of the modules have an average Zsummary. pres statistic larger than 4.4 and the average Zsummary. pres statistic of all modules was 12.4, indicating very strong preservation of modules except GSE89632 which has weak to moderate preservation Zsummary. pres statistic.

### Modules are correlated with clinical parameters

**FIGURE 4:**
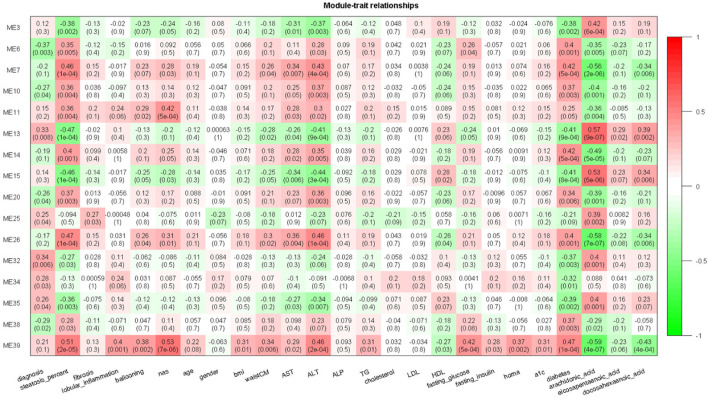
Module-trait relationship heatmap for GSE89632. Cells represent the correlation between modules expression and clinical parameters. Numbers in the bracket indicate the statistical significance.

**FIGURE 5:**
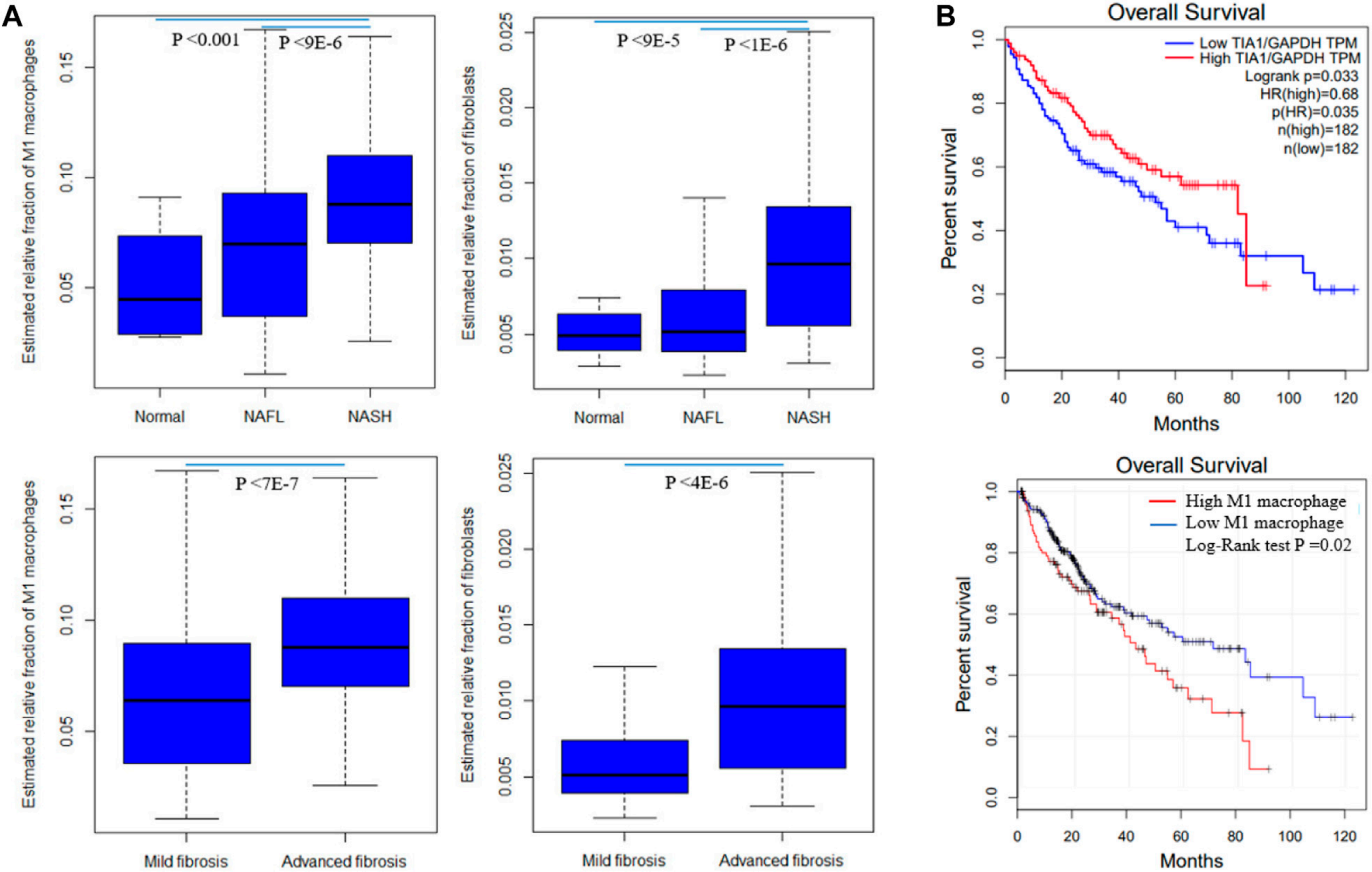
Fibroblast and M1 macrophage in NAFLD and HCC. **(A)** Differentially expressed fractions of M1 macrophages and fibroblasts in patients with different NAFLD stages and fibrosis stages in GSE135251 **(B)** M1 macrophage and hub gene *Tia1* are associated with HCC patients survival in the TCGA dataset. *Tia1* is the hub gene of M15 which is negatively correlated with M1 macrophage proportion. The log-rank derived *p*-value indicates the significance of the comparison between groups. Statistical significance was calculated by Student’s t-test for mild and advanced fibrosis comparison, or ANOVA for normal, NAFL, and NASH comparison.

By correlating modules with clinical parameters, we can identify which module contributes to disease. Module-trait relationship analysis revealed several connections. M25 is positively correlated with fibrosis in all six datasets that provide fibrosis data (Supplementary Table S2). As dataset GSE89632 has abundant clinical parameters, thus we correlated module expression and these parameters in the dataset. M39 and M35 are positively and negatively correlated with hepatic fat content in two datasets. M39 is positively correlated with NAFLD activity score (NAS), fasting glucose, and diabetes status in GSE89632. M13 and M39 are positively and negatively correlated with arachidonic acid (Figure 4). Module-trait relationship results for the other datasets are provided in Supplementary Figure S1. We also performed TIMER analysis to estimate the immune cell populations in the liver tissues of patients with NAFLD dataset GSE135251. Fibroblasts and M1 macrophages are positively correlated with fibrosis. The correlation coefficients are 0.42 and 0.35, and the *p* values are 2E-10 and 1E-7 respectively. Further analysis shows that the two components are upregulated during NAFLD progression. Patients with NASH and advanced fibrosis had greater fractions of fibroblasts and M1 macrophages than patients with NAFL and mild fibrosis (Figure 5A). The fraction of M2 macrophages is downregulated during NAFLD progression but not significant. NAFLD-related HCC has been reported in a significant number of patients (Margini and Dufour, 2016). Thus, we also analyzed the two components in the TCGA HCC dataset and found that low M1 macrophage fraction group patients had longer overall survival (Figure 5B). M15 is negatively correlated with M1 macrophage proportion (Table 2). M15 hub gene TIA1 expression suggests a positive contribution to HCC patient survival (Figure 5B).

### Differentially expressed modules associated with steatosis, fibrosis, and steatohepatitis

**FIGURE 6:**
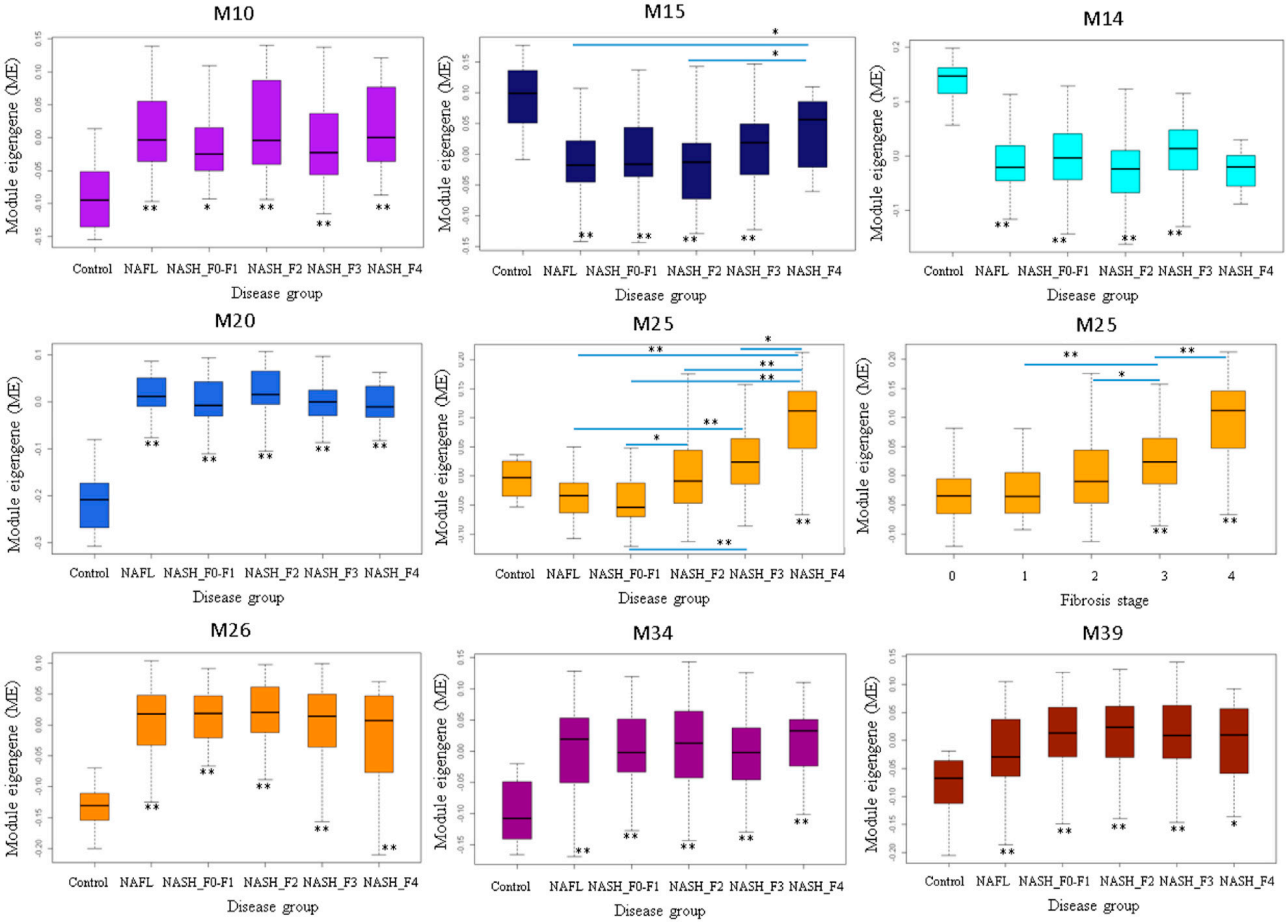
Differentially expressed modules associated with steatohepatitis stages in GSE135251. The asterisk under the box indicates the significance of the comparison between disease and normal control by ANOVA test with adjusted *p* values. *: *p* < 0.05, **: *p* < 0.01.

**FIGURE 7:**
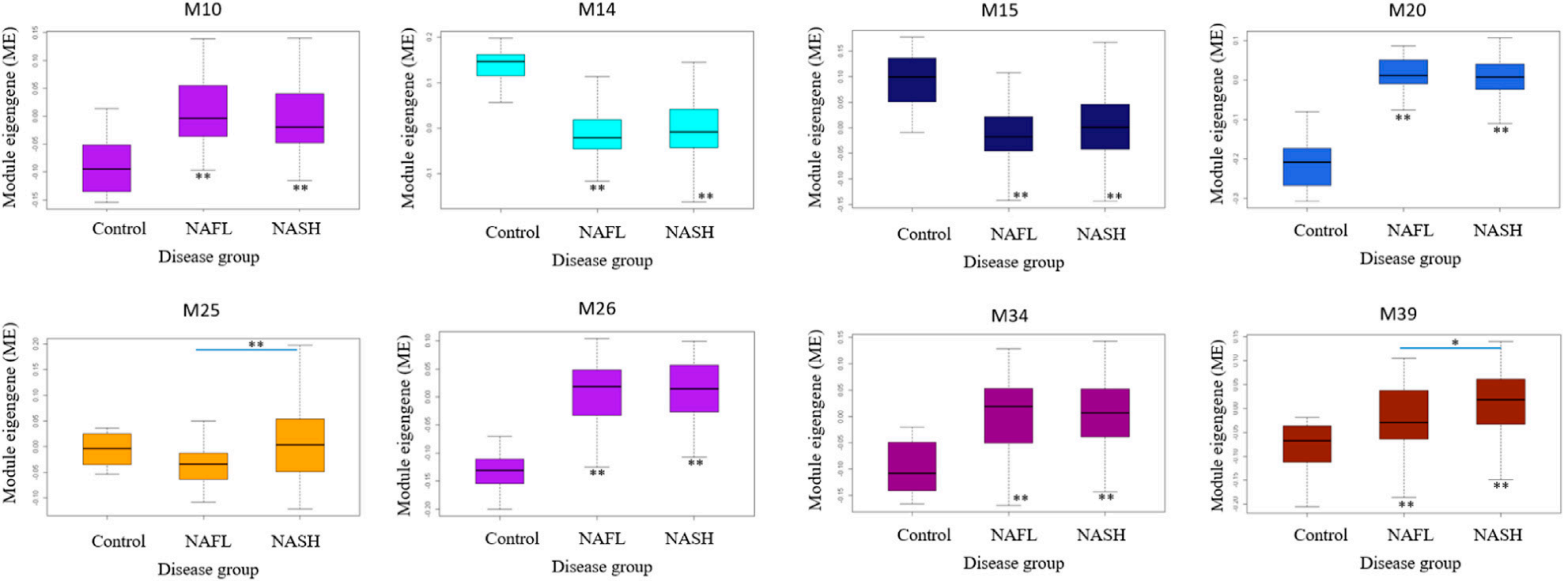
Differentially expressed modules associated with NAFLD disease stages in GSE135251. The asterisk under the box indicates the significance of the comparison between disease and normal control by ANOVA test with adjusted *p* values. *: *p* < 0.05, **: *p* < 0.01.

M25 was consistently upregulated in advanced fibrosis compared to mild fibrosis in five datasets (*t*-test, *p* < 0.05). In GSE135251 only M25 was differently expressed across fibrosis stages (Figure 6). M14 and M20 were the most significantly upregulated in NAFL samples compared to the control. M25 and M39 were the only two differential modules upregulated in NASH compared to NAFL (*p* = 0.0003 and *p* = 0.02). M25 and M14 were the most significantly upregulated and downregulated in NASH samples compared to no NASH (*p* = 0.001 and *p* = 0.04) (Figure 7). The box figures for all the modules can be viewed in the “Differential module analysis” tab of the NAFLD database at http://nafld.shinyapps.io/shiny/.

### Modules can consistently separate NAFLD patients

To check if these modules can efficiently separate patients, we calculated specificity and sensitivity to plot the receiver operating characteristic (ROC) curve. Modules such as M20, M26, and M39 can recurrently separate control and NAFL patients in different datasets with AUCs ranging from 0.76 to 1.00 (Supplementary Figure S2). M20, M25, M26, M32 can separate samples from NAFL and NASH with AUCs larger than 0.88 (Supplementary Figure S3). M25 could consistently separate samples from mild fibrosis and advanced fibrosis in six datasets with AUCs 0.89, 0.96, 0.97, 0.84, 0.82, and 0.76. (Supplementary Figure S4).

### Hub genes are important in NAFLD progression

**FIGURE 8:**
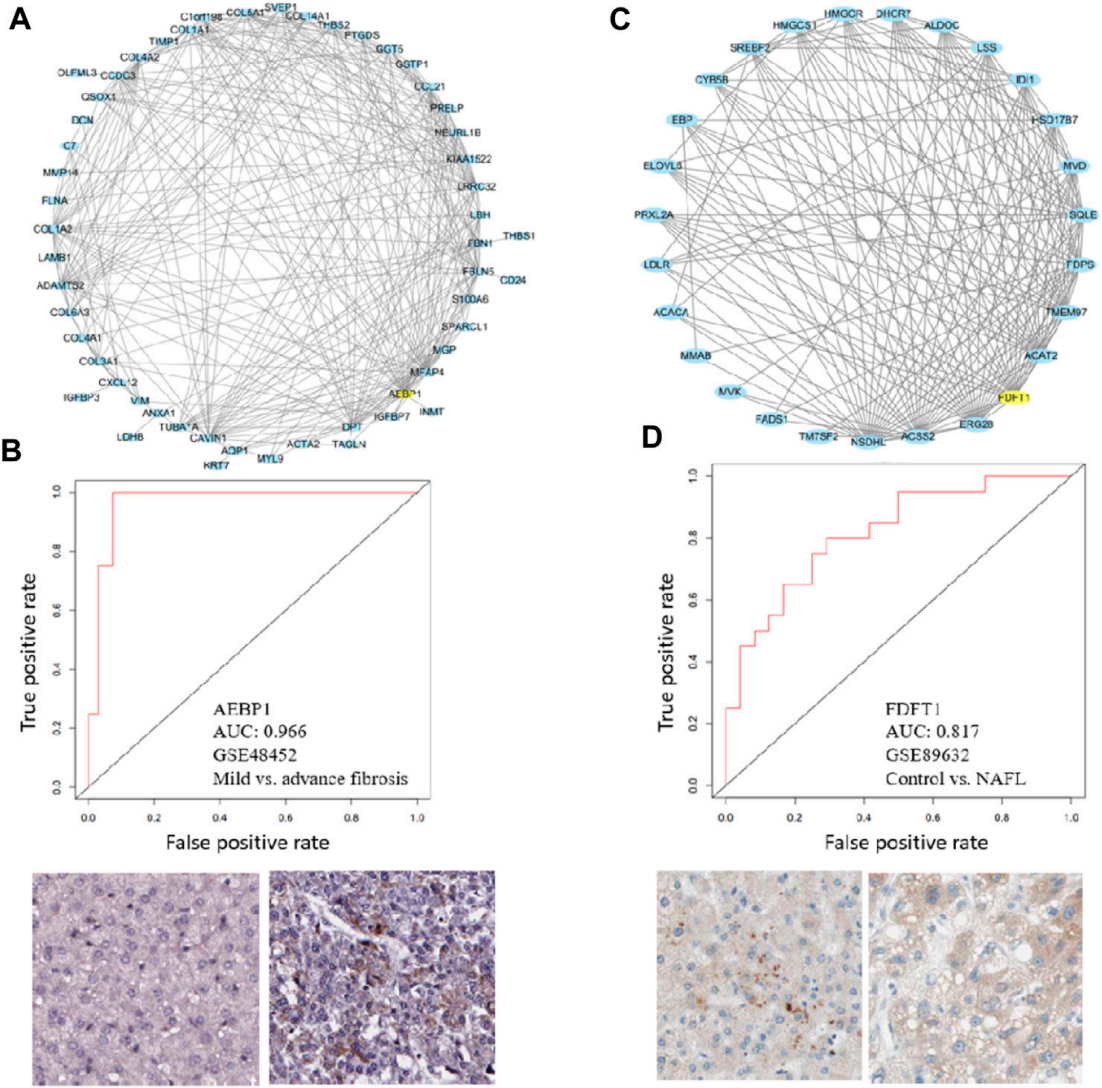
Hub genes in M25 and M39 can separate patients. **(A, C)** Module visualization for the top 100 connections within M25 and M27 by using Cytoscape. Yellow nodes indicate the hub gene of modules **(B, D)** ROC curves show that hub genes of M25 and M39, *Aebp1*, and *Fdft1* can separate advanced fibrosis or NAFL patients. Immunohistochemical plots also show the positive staining of AEBP1 and FDFT1 in normal and HCC tissues.

To demonstrate the utility of the co-expression modules identified, two important modules M25 and M39 were visualized. *Aebp1* is the hub gene of M25 (Figure 8A). It has been reported that adipocyte enhancer-binding protein 1 (AEBP1) is a ubiquitously expressed, multifunctional protein and is a transcriptional repressor involved in adipogenesis, inflammation, cholesterol homeostasis, and atherogenesis (Majdalawieh et al., 2020). AEBP1 expression increases with the severity of fibrosis in NASH possibly by encoding the aortic carboxypeptidase-like protein (ACLP) that associates with collagens in the extracellular matrix (ECM) (Blackburn et al., 2018; Gerhard et al., 2019). In our analysis, M25 indeed is associated with fibrosis, and *Aebp1* is highly correlated with ECM genes *Col1a1, Col1a2, Col3a1, Col4a1, Col4a2, Col5a1, Col6a3, And Col14a1* (Figure 8A). *Aebp1* has a good performance in separating mild and advanced fibrosis patients (Figure 8B). *Fdft1* is the hub gene of M39 (Figure 8C). *Fdft1* is one of the causative loci for steatosis, NAS, degree of fibrosis, lobular inflammation, and serum levels of alanine aminotransferase (ALT) (Stattermayer et al., 2014; Sharma et al., 2015). Interestingly, we found that M39 is highly correlated with steatosis, NAS, lobular inflammation, ALT, and diabetes (Figure 4). *Fdft1* has a moderate performance in separating healthy control and NAFL patients (Figure 8D). The two proteins were positively stained in HCC liver tissues. As hepatic fibrosis presents in a majority of HCC patients, we also checked the two gene expression in TCGA pan-cancer datasets and found that the two genes were not liver-specific genes and were not prognostic for HCC overall survival (Supplementary Figure S5). All the module information can be explored at the NAFLD co-expression database at http://nafld.shinyapps.io/shiny/, where users can browse gene lists, hub genes, functional annotation, network, correlation analysis, and differential module expression information.

### Correlation between modules and enzymes regulating m^6^A mRNA methylation

N6-methyladenosine (m^6^A) modification contributes to metabolic reprogramming in NAFLD (Qin et al., 2021). To discover potential m^6^A regulators of modules, we selected 24 related genes within the dataset and correlated them with MEs by Spearman correlation. Some of the m^6^A genes were highly correlated with module expression. For example, YTHDF3, YTHDC2, METTL14, CBLL1, and M7 all had significant correlations larger than 0.75. YTHDF3, YTHDC2, METTL14, CBLL1, and M10 had significant correlations smaller than −0.70. FTO-M26, Eif3A-M35, and VIRMA-M35 all had significant correlations larger than 0.70 (Supplementary Table S3). These results were consistent with the reverse correlations with Th1 CD4 T cells between M7 and M10. M26 was correlated with macrophage proportion, which is important in NASH. *Fto* is correlated with M26, indicating the role of *Fto* in NASH progression.

### *In silico* drugs screening based on highly connected genes in differentially expressed modules

**FIGURE 9:**
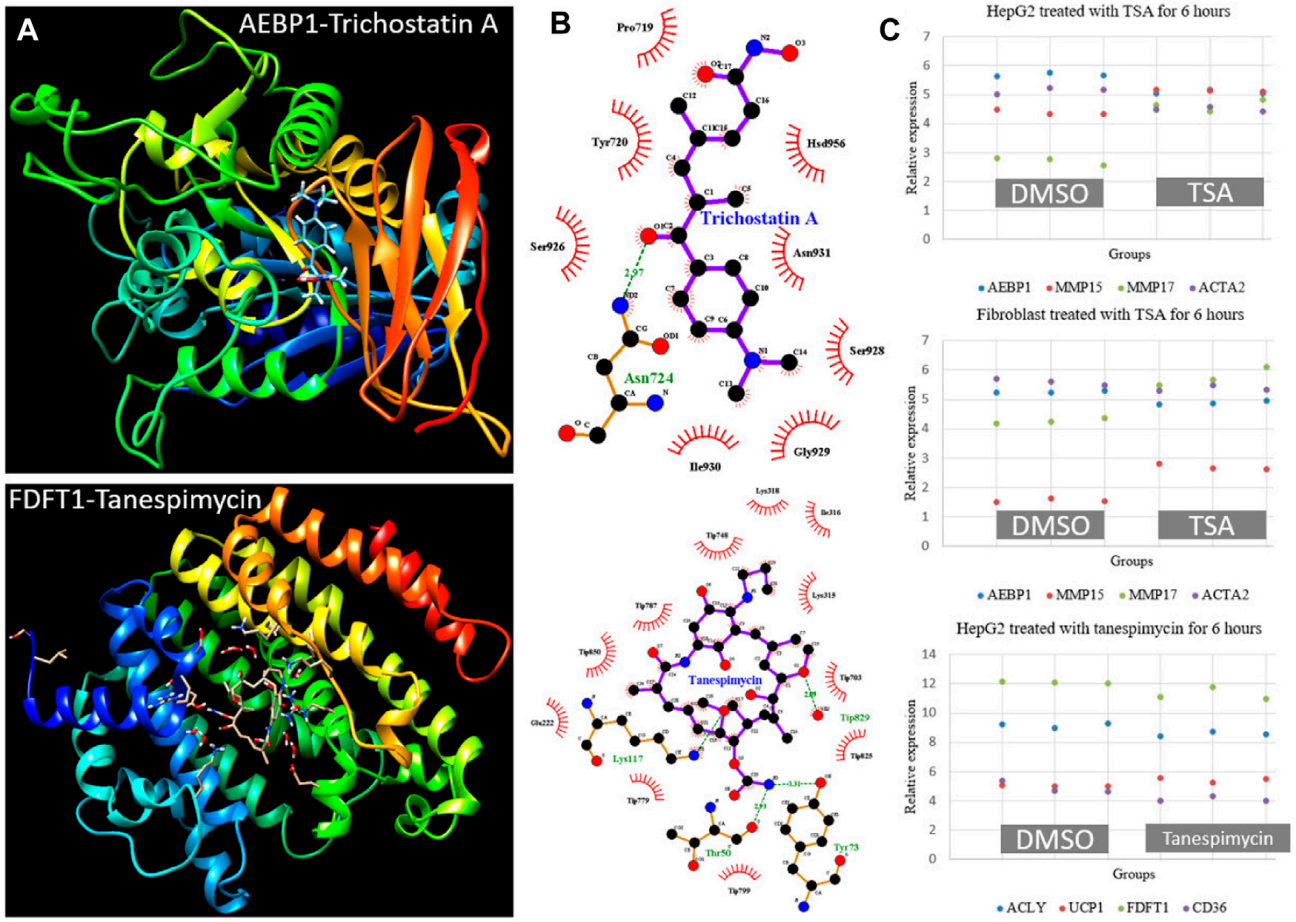
Protein-ligand docking analysis and expression validation for hub genes AEBP1 in M25 and FDFT1 in M39. **(A)** 3-D structure models for AEBP1-Trichostatin A and FDFT1-Tanespimycin **(B)** Pose view for the interaction sites. The hydrogen bonds were visualized in the green dashed line **(C)** AEBP1 and fibrosis-related genes MMP15, MMP17, and ACTA2 expression changed after TSA treatment in HepG2 and fibroblast cell lines from dataset GSE200186. FDFT1 and lipid metabolism-related genes ACLY, UCP1, and CD36 expression changed after tanespimycin treatment in HepG2 from dataset GSE119281. All the gene expression was detected with three replicates and had a *p* < 0.05 by Student’s t-test.

To identify candidate drugs for NAFLD, we selected ten genes from differentially expressed modules and submitted them to the Connectivity Map tool. As listed in Table 3, eight candidates were found with *p* < 0.05. Protein-ligand docking analysis showed the interactions between hub genes *Aebp1* (M25), *Fdft1* (M39) and Trichostatin A (TSA), Tanespimycin (Figure 9A). Pose view analysis showed that the interactions may occur at sites Asn724 in AEBP1 and Lys117, Thr50, Tyr73 in FDFT1 (Figure 9B). Independent datasets were used to validate the results. Interestingly, TSA can significantly downregulated *Aebp1* in HepaG2 and fibroblast cell lines. Additionally, the expression of fibrosis-related genes *Mmp15, Mmp17,* and *Acta2* also changed after 6 h TSA treatment (Figure 9C). As for tanespimycin, *Fdft1* was significantly downregulated after treatment in HepaG2. Additionally, the expression of genes *Acly* (lipogenic gene), *Ucp1* (thermogenic gene), and *Cd36* (lipid uptake) also changed after 6 h tanespimycin treatment (Figure 9C). All the candidate drugs are worth validation in future studies.

**TABLE 3:** Candidate NAFLD drugs identified by Connectivity Map.

No.	Drug	*p*-value
1	Trichostatin A	2E-7
2	Geldanamycin	4E-4
3	Tanespimycin	0.015
4	Diphenylpyraline	0.024
5	6-bromoindirubin-3′-oxime	0.024
6	Pha-00851261E	0.028
7	Lisuride	0.028
8	Clebopride	0.029

## Discussion

We collected a comprehensive compendium of NAFLD transcriptome datasets. Traditional transcriptome data analysis is based on differential analysis methods. However, the number of sample groups in a study may not just be two, if considering different fibrosis stages or other parameters. The subject size varies in studies. Thus, it is somewhat hard to reach a favorable overlap of differential genes between studies. Only four genes, namely, *Col1a2, Efemp2, Fbln5*, and *Thbs2* were found consistently across six published transcriptomic studies on NASH(Pantano et al., 2021). There can be many reasons for the low overlap, such as the inherent complexity of the liver which is composed of heterogeneous cell types, different biopsy sites or methods, different sample preparation workflows, and different transcriptome profiling platforms. We turned to the module-centric analysis, as the module represents a high level of the regulatory scenario (Wang et al., 2008). Several genes may participate in a common biological function. The gene change in one dataset may not replicate in other datasets, but the biological function may replicate in other datasets. We used one dataset for reference module identification, and verify modules in the other 10 datasets. Results suggest that gene co-expression networks from microarray and RNA-Seq are generally reproducible. Based on these modules, we can correlate modules with different clinical data from different studies.

We identified several biological processes that have been known in NAFLD progressions, such as mitochondrion, endoplasmic reticulum, collagen, sterol biosynthesis, and leukocyte activation (Simoes et al., 2018). Some of the identified modules do not have obvious function annotations, such as M35. In our analysis, M35 was downregulated in NAFL compared to normal (GSE89632) and in higher-grade fibrosis compared to control (GSE162694 and GSE130970). Recent reports suggest that activated hsa-miR-21–5p can promote steatosis and fibrosis (Calo et al., 2016; Tadokoro et al., 2021). The Hub gene of M35 *Dennd4c* has not been reported in NAFLD. Thus, it can serve as a potential therapeutic target in future studies. The Hub gene of M38 *Stoml2* is also currently not reported to be associated with NAFLD. In our analysis, M38 was downregulated in NAFL compared with the control. Therefore, it can serve as a potential therapeutic target for steatosis in future studies.

In our module-based analysis, the AUCs for predicting NAFL and advanced fibrosis were 0.93 (M20) and 0.89 (M25), respectively, which were comparable to a recent report (Kozumi et al., 2021). The M20 hub *Clptm1* has a perfect performance (AUC = 0.99) in separating NAFL and normal control. CLPTM1 is located in the endoplasmic reticulum (ER) and is involved in ER stress (Liu et al., 2018b). M20 is the most significant differential module in the spectrum of NAFLD, indicating the important role of ER in disease progression.

One of the important findings in this study is that the gene co-expression modules can efficiently separate NAFLD patients. Although signature genes have been proposed for patient stratification, few genes showed good reproducibility across studies (Vandel et al., 2021). The present study incorporates data from a large number of NAFLD patients and has been validated with multiple databases, showing a satisfactory performance in NAFLD prognosis. In addition, the hub genes of the two modules can be used as biomarkers for NAFLD patient stratification.

We also found that modules were correlated with NAFLD clinical features. As the number of clinical features is variable across datasets, we focused on the common correlations. It may be hard to explain the differences between datasets, as the reason for differences is hard to trace. Correlations detected in more than two datasets may be more reliable module-trait associations. Besides modules M39 and M35, we found M32 was associated with fibrosis and NAS in four datasets (Supplementary Figure S1).

Finally, we proposed drug repurposing by analyzing highly connected genes in the Connectivity Map tool. Some of the identified drugs have been indicated in roles of fibrosis, inflammation, or fat metabolism although not in the liver. For example, TSA is an HDAC inhibitor that could alleviate atrial fibrosis and subsequent atrial fibrillation (Liu et al., 2008). It also can reduce systemic inflammation and improve the survival of the mice model of sepsis (Cui et al., 2019). 17-AAG also known as tanespimycin, a derivative of the antibiotic geldanamycin that has a higher affinity to HSP90, could inhibit fibroblast activation and reduce ECM production (Sontake et al., 2017). Tanespimycin is an Hsp90 inhibitor that can prolong survival, attenuate inflammation, and reduce lung injury in mouse models of sepsis (Chatterjee et al., 2007). Thus, these drugs are promising candidates for the treatment of NASH. Only a recent study showed that antidepressants such as diphenylpyraline could activate FAM3A to suppress hepatic gluconeogenesis and lipogenesis, finally improving hyperglycemia and steatosis in obese diabetic mice (Chen et al., 2020). It was found that inhibition or downregulation of the canonical Wnt/β-catenin pathway contributes to the disease progression of NAFLD (Shree Harini and Ezhilarasan, 2022). While Glycogen synthase kinase 3 (GSK3) inhibitors could activate canonical Wnt/β-catenin signaling and then promote hepatocyte differentiation (Huang et al., 2017). In an animal model, it was proved that GSK3 inhibitor 6-bromoindirubin-3′-oxime (6BIO) could modulate bioenergetic pathways and decrease lipid and glucose tissue load (Tsakiri et al., 2017). Thus, the two drugs are promising candidates for future validation.

However, there are still some limitations to our study. WGCNA generates an undirected network, lacking information on the regulation direction between genes. The collected transcriptome is still not large enough, and some lack detailed clinical information, making the integration of datasets from studies difficult. The identified modules can be used as biomarkers for prognosis, but not sufficiently to reveal of disease mechanism. More work is needed to validate upstream genes that control the co-expression of modules.

## Data Availability

Publicly available datasets were analyzed in this study. This data can be found here: The datasets analyzed in the study are available in public repositories at NCBI GEO (https://www.ncbi.nlm.nih.gov/geo/) and EBI ArrayExpress (https://www.ebi.ac.uk/arrayexpress/).
